# Descriptive Epidemiology of Typhoid Fever during an Epidemic in Harare, Zimbabwe, 2012

**DOI:** 10.1371/journal.pone.0114702

**Published:** 2014-12-08

**Authors:** Jonathan A. Polonsky, Isabel Martínez-Pino, Fabienne Nackers, Prosper Chonzi, Portia Manangazira, Michel Van Herp, Peter Maes, Klaudia Porten, Francisco J. Luquero

**Affiliations:** 1 Epicentre, Paris, France; 2 European Programme for Intervention Epidemiology Training, European Centre for Disease Prevention and Control, Stockholm, Sweden; 3 Ministry of Health and Child Welfare, Harare City Health Department, Harare, Zimbabwe; 4 Ministry of Health and Child Welfare, Epidemiology and Disease Control Directorate, Harare, Zimbabwe; 5 Médecins Sans Frontières Operational Centre Brussels, Brussels, Belgium; The Australian National University, Australia

## Abstract

**Background:**

Typhoid fever remains a significant public health problem in developing countries. In October 2011, a typhoid fever epidemic was declared in Harare, Zimbabwe - the fourth enteric infection epidemic since 2008. To orient control activities, we described the epidemiology and spatiotemporal clustering of the epidemic in Dzivaresekwa and Kuwadzana, the two most affected suburbs of Harare.

**Methods:**

A typhoid fever case-patient register was analysed to describe the epidemic. To explore clustering, we constructed a dataset comprising GPS coordinates of case-patient residences and randomly sampled residential locations (spatial controls). The scale and significance of clustering was explored with Ripley K functions. Cluster locations were determined by a random labelling technique and confirmed using Kulldorff's spatial scan statistic.

**Principal Findings:**

We analysed data from 2570 confirmed and suspected case-patients, and found significant spatiotemporal clustering of typhoid fever in two non-overlapping areas, which appeared to be linked to environmental sources. Peak relative risk was more than six times greater than in areas lying outside the cluster ranges. Clusters were identified in similar geographical ranges by both random labelling and Kulldorff's spatial scan statistic. The spatial scale at which typhoid fever clustered was highly localised, with significant clustering at distances up to 4.5 km and peak levels at approximately 3.5 km. The epicentre of infection transmission shifted from one cluster to the other during the course of the epidemic.

**Conclusions:**

This study demonstrated highly localised clustering of typhoid fever during an epidemic in an urban African setting, and highlights the importance of spatiotemporal analysis for making timely decisions about targetting prevention and control activities and reinforcing treatment during epidemics. This approach should be integrated into existing surveillance systems to facilitate early detection of epidemics and identify their spatial range.

## Introduction

Typhoid fever, a systemic infection caused by *Salmonella enterica* serotype Typhi (*S.* Typhi), is an important cause of morbidity and mortality globally [1]–[4]. It remains a major public health problem in developing countries, where access to clean water and standards of sanitation and hygiene are inadequate [2], [3], [5]. Recent reviews have estimated the annual global number of episodes of disease caused by *S*. Typhi at between 21.6 and 26.9 million, with more than 216 000 deaths globally in 2000 [1], [4]. Humans are the only natural host and reservoir for *S.* Typhi, which can survive several months in soil or water [2], [6]. The infection is transmitted by ingestion of food or water contaminated by faecal or urinary carriers excreting the bacterium [5]. Little is known about the transmission dynamics of typhoid fever, beyond an association between declining incidence with improvements in sanitation and risk factors identified through classical epidemiological techniques, particularly case-control studies [3], [7], [8]. Individual-level risk factors are, among others, contaminated water supply, food bought from street vendors, the consumption of raw fruit and vegetables, and a history of contact with other cases or chronic carriers; community-level risk factors include population density, temperature, rainfall, river level, and proximity to water sources [2], [6], [9]–[12]. Blood culture is the mainstay for diagnosing typhoid fever [5], [13].

Harare, the capital of Zimbabwe, experienced several enteric infection epidemics in recent years: in 2008 and 2009, and again in 2010, epidemics of cholera affected large parts of the country [14], [15], while in 2010, Harare experienced an epidemic of typhoid fever [15]. These epidemics have been linked to chronic underinvestment in the maintenance of water and sanitation infrastructure, leading to irregular water supplies, difficulties in protecting drinking-water supplies, and the breakdown of sanitation systems [14], [16].

On 10 October 2011, the Zimbabwean Ministry of Health declared a new epidemic of typhoid fever in Harare. Médecins Sans Frontières-Operational Centre Brussels (MSF) supported the Ministry in responding to the epidemic. MSF, in collaboration with the Ministry, used surveillance data and geographical information system data collected by community health workers to describe the epidemic and map its spatiotemporal distribution within Dzivaresekwa and Kuwadzana, the two worst affected suburbs. Spatial techniques have been used increasingly to describe clustering of infectious and non-infectious diseases: this has been done in few instances to identify the spatial distribution of, and risk factors associated with, typhoid fever, and only in Asia [7], [9], [12], [17], [18] and the USA [19]. This article describes such approaches and their effectiveness for identifying foci of transmission in an urban African context.

## Methods

### Ethics statement

Specific ethical approval for this study was not sought as it formed part of emergency response surveillance activities initiated for epidemic control. The confidentiality and rights of patients were ensured during and after the conduct of the study. The investigation was implemented in collaboration with the City Health Department of Harare, after authorization from the Ministry of Health and Child Welfare.

### Descriptive epidemiology

An individual register of suspected and confirmed cases was maintained by the City Health Department and shared with MSF. This register included information on age, sex, symptoms onset date, residential address and clinical outcome, which was used to describe the epidemic in terms of time, place and person.

A modified WHO clinical case definition was used for suspected and confirmed cases of typhoid fever [20]. A suspected case was defined as any person with gradual onset of steadily increasing and then persistently high fever, chills, malaise, headache, sore throat, cough, and, sometimes, abdominal pain and constipation or diarrhoea. A confirmed case was defined as a suspected case confirmed by isolation of *S.* Typhi from blood, bone marrow, bowel fluid or stool. Blood, stool and urine samples were analysed at the Beatrice Road Infectious Disease Hospital in Harare to confirm cases and to determine the circulating strain.

Suburb- and age-specific attack rates (the percentage of the population who were suspected case-patients) were estimated. For these calculations, suburb- and age-specific population figures were calculated from the 2002 official census of Harare. To estimate the population at the time of the epidemic, we multiplied these figures by an annual average growth rate of 3%, as estimated by the Population Division of the Department of Economic and Social Affairs of the United Nations [21].

### Spatial analysis

The retrospective mapping was limited to Dzivaresekwa and Kuwadzana suburbs. Trained community health assistants used handheld global positioning system (GPS) devices (Garmin eTrex HC) to record the coordinates of the residences of case-patients listed in the individual register. The locations of a convenience sample of water sources (including boreholes, taps and wells) were obtained from the Centers for Disease Control and Prevention [22] in a study conducted during the epidemic. Each case-patient in the line list was attributed one point, with multiple case-patients within a household being assigned one point each.

For the control points, we wrote R [23] code to generate random GPS points in each of Dzivaresekwa and Kuwadzana suburbs, the geographical boundaries of which were delimited by shapefiles. This code (see S1 Supporting Information) generated sequential points at random locations within the shapefile boundaries. We generated one random control point for each case-patient in the line list whose address was successfully traced and recorded (N = 2212); the final number of points generated in each suburb was proportional to its relative population size (949 in Dzivaresekwa and 1263 in Kuwadzana) to ensure a relatively homogenous distribution of control points within residential areas. We generated sequential points until we reached this desired sample size (949 and 1263), having retained only those points that coincided with the location of a residential structure when visualised in Google Earth (Google 2013). These controls were used to adjust the spatial estimate of typhoid fever risk for the heterogeneous spatial distribution of residences in the study area (by K function difference, random labelling, and Kulldorff's spatial scan statistic), according to the methods detailed below.

We used a ‘case-control’ approach to detect spatial clustering (areas in which there was significant spatial grouping of events), in which ‘cases’ (case-patients) represented the spatial risk of the disease and ‘controls’ (computer-generated random points) represented the geographical distribution of the population in the study area. We constructed a spatial point pattern dataset comprising the locations of case-patients and control points, which we represented on a map. The intensity of cases and controls (number of events per square kilometre) was calculated by a Kernel smoothing technique; the mean square error of the Kernel was used to select the Kernel bandwidth parameter [24]–[26]. Briefly, the Kernel smoothing technique involves a three-dimensional function (the kernel), which scans an area of interest, estimating the intensity of events within a ‘sphere of influence’ (the size of which is defined by the ‘bandwidth’) at each location, and weighting these according to their distance from the point being estimated [27]. The result is a smoothed surface, the height of which at any given point is an estimation of the intensity of the event.

The scale and significance of geographical clustering was explored with Ripley *K* functions [28], in a method described in detail elsewhere [27]. Briefly, *K* functions describe the extent to which there is spatial dependence in the arrangement of events of the same type; where there is clustering of point events, an excess of events at short distances is observed [27], [29]. *K* functions can be plotted such that *K*(s) appears on the y axis against distance on the x axis. Variations in the spatial distribution of the population are, however, likely to cause all population-based events, including cases of disease, to cluster independently of any other processes, such as disease transmission dynamics [27]. In order to correct for the spatial distribution of residences in the study area, we constructed a model to infer the spatial risk of *S.* Typhi infection by comparing the *K* function calculated for case-patients with a *K* function calculated for the randomly generated control points. The difference between the two functions, Dhat(s), represents a measure of the extra aggregation of one *K* function over the other [24], [27], [29]. We performed 1000 Monte-Carlo simulations to generate a 95% confidence envelope for Dhat(s); clustering was judged to have occurred over distances for which Dhat(s) exceeded this simulated envelope, at a significance threshold of p = 0.05.

We estimated the spatial variation log risk ratio for typhoid fever, representing the strength of clustering of case-patients relative to control points. We used the cross-validation method to select the common bandwidth parameter of the Kernel when comparing case-patients and control points [30], [31]. This technique was applied for all weeks and also by the onset of symptoms to provide an overview of the epidemic disaggregated by time. The weekly data were used to construct a movie file showing the evolution of the log risk ratio over the course of the epidemic (S1 Video). We assessed the significance of the clustering by a random labelling technique, using 1000 Monte-Carlo simulations to derive upper and lower confidence limits [32], [33]. Kulldorff's spatial scan statistic, an explicit test for the presence of clusters, was used to confirm the clustering [34]–[36]. Briefly, it calculates local rates of disease inside scanning circles of various sizes by placing circles at each case and control point and calculating a likelihood ratio test of potential clusters by comparing the null hypothesis (that the disease risk is equal inside and outside the circle) with the alternative hypothesis (that there is an elevated disease risk inside the circle); circles with maximum likelihood are the most likely clusters [35], [37].

All analyses were performed with R version 2.15.0 [23].

## Results

### Descriptive epidemiology

**Figure 1:**
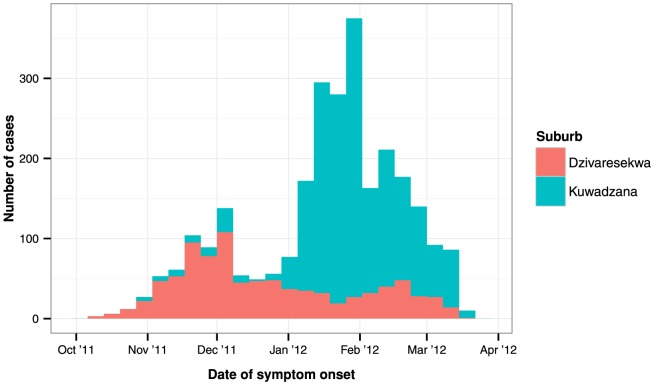
Weekly number of suspected cases of typhoid fever, by date of symptom onset, registered in Harare, 10 October 2011–17 March 2012.

The first case-patient of typhoid fever detected during this epidemic was reported on 10 October (week 41) 2011. As of 17 March 2012, a total of 3795 cases of typhoid fever had been reported, of which 62 (1.2%) were confirmed by laboratory diagnosis. Two deaths were reported, corresponding to a case fatality ratio of 0.05%. Dzivaresekwa and Kuwadzana accounted for 2570 (67.7%) case-patients. In these suburbs, two phases of the epidemic were distinguishable: the first occurred in Dzivaresekwa and peaked in late November 2011, while the second occurred in Kuwadzana and peaked in early February 2012 (Fig. 1).

The attack rates in Dzivaresekwa and Kuwadzana were 0.66 and 1.13%, respectively, and 0.93 overall (Table 1). The median age of the 2570 case-patients in Dzivaresekwa and Kuwadzana was 15 years (interquartile range: 4–29 years). There was a trend of decreasing age-specific attack rate with increasing age. Females experienced a higher attack rate than males and accounted for 1428 (55.6%) of patients. Most case-patients included in the analysis were the first ones detected at their residential addresses, while 135 (5.3%) case-patients included in the analysis were subsequent case-patients detected at their residential address.

**Table 1:** Numbers of suspected cases of typhoid fever, estimated population size, and attack rates by suburb, gender, and age group, in Dzivaresekwa and Kuwadzana suburbs, Harare, Zimbabwe, 10 October 2011–17 March 2012.

Variable	Cases (%)	Population^1^	Attack rate (%)
**Suburb**			
Dzivaresekwa	785 (30.5)	118 271	0.66
Kuwadzana	1 785 (69.5)	157 396	1.13
Gender*			
Male	1 142 (44.4)	138 204	0.83
Female	1 428 (55.6)	137 463	1.04
**Age (years)**			
0–4	693 (27.0)	36 663	1.89
5–14	560 (21.8)	51 550	1.09
15–49	1 160 (45.1)	160 990	0.72
>50	157 (6.1)	26 464	0.59
**Total**	2 570 (100)	275 667	0.93

1 Based on the 2002 census, to which we applied a correction factor of 3% per year to account for estimated annual growth.

### Spatial analysis

Between 7 and 17 March 2012, we traced and recorded the GPS coordinates of the residential addresses of 2212 (86.1%) of the case-patients recorded in the line list: 697 (31.5%) in Dzivaresekwa and 1515 (68.5%) in Kuwadzana. Only these case-patients were included in the spatial analysis.

**Figure 2:**
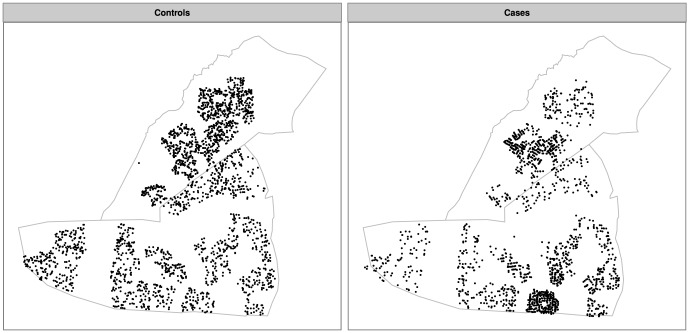
Geographical distribution of control points (representing the distribution of residential areas) and of suspected cases of typhoid fever in Dzivaresekwa and Kuwadzana suburbs, Harare, Zimbabwe, 10 October 2011–17 March 2012.

**Figure 3:**
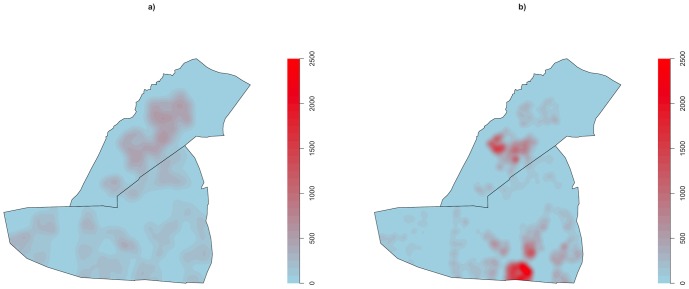
Intensities of a) control points (representing the distribution of residential areas) and of b) suspected cases of typhoid fever, measured as events per km^2^, in Dzivaresekwa and Kuwadzana suburbs, Harare, Zimbabwe, 10 October 2011–17 March 2012.

Fig. 2 shows the raw geographical distribution of control-points (‘Controls’) and case-patients (‘Cases’), while Fig. 3 shows the primary mapping of the *intensities* of these control points (Fig. 3a) and case-patients (Fig. 3b). Fig. 3a reveals the heterogeneous distribution of residences throughout Dzivaresekwa and Kuwadzana, with an intensity of control points of 0–780 points per km^2^. Case-patients were concentrated in two areas (one in each of the two suburbs), with intensity between 0 and approximately 2500 case-patients per km^2^.

**Figure 4:**
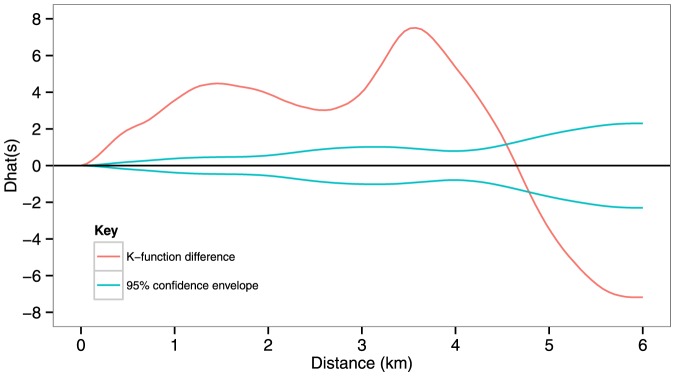
Differences of K functions (red line) and 95% confidence envelope (blue lines) between suspected cases of typhoid fever and controls in Dzivaresekwa and Kuwadzana suburbs in Harare, Zimbabwe, 10 October 2011–17 March 2012.

The difference between the *K* functions for case-patients and control points at varying distances (Fig. 4) revealed significantly more clustering of case-patients for distances up to approximately 4.5 km than for the underlying population (p<0.001). Two peak differences occurred, at approximately 1.5 and 3.5 km.

**Figure 5:**
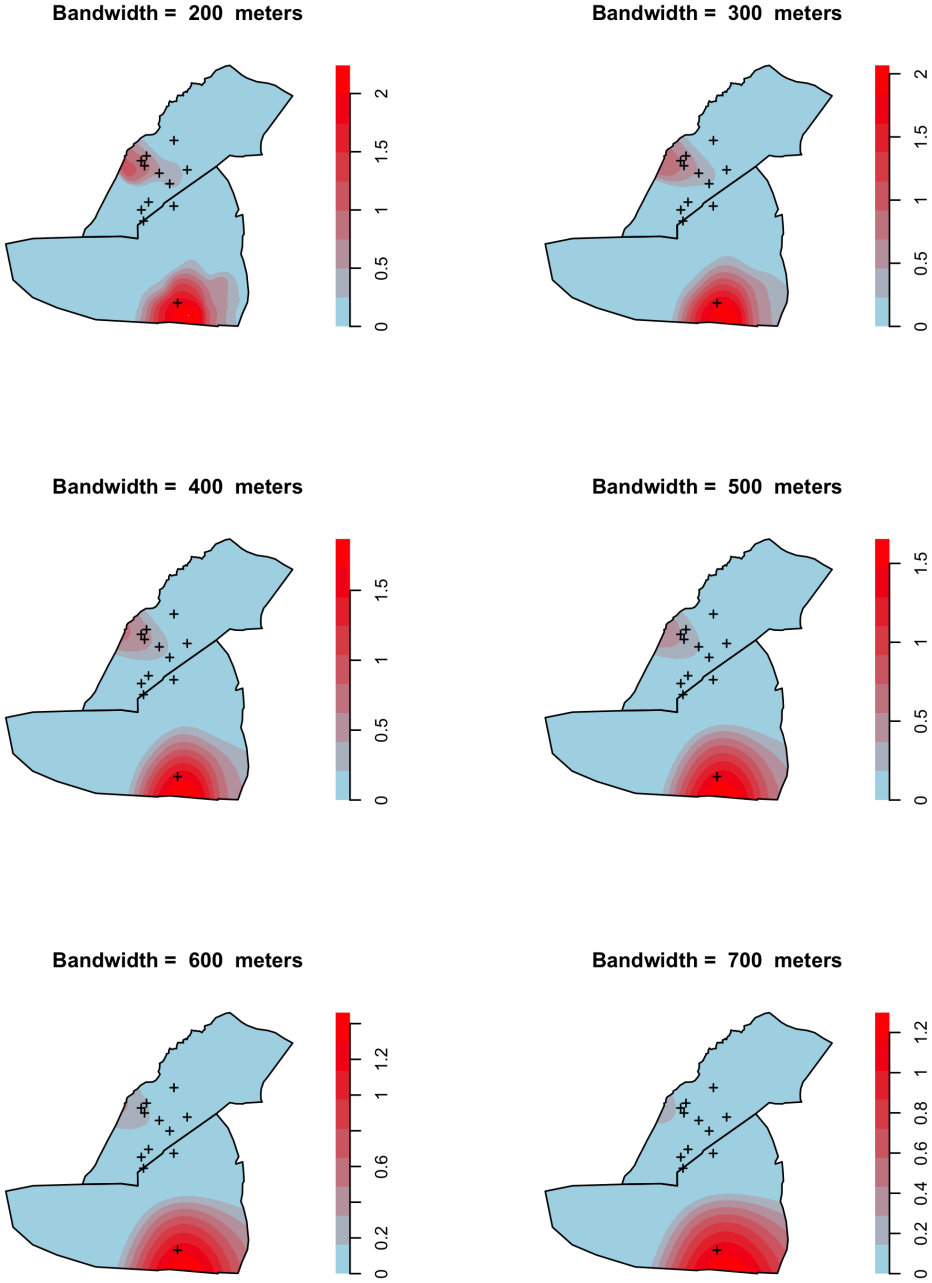
Geographical distribution of the log relative risk of typhoid fever in Dzivaresekwa and Kuwadzana suburbs in Harare, Zimbabwe, October 10 2011–March 17 2012, according to different bandwidths used for calculating the kernel function.

Fig. 5 shows the log relative risk for typhoid fever throughout the epidemic, with different bandwidths used for calculating the kernel function. This series of maps shows that the locations of the clusters are generally unaffected by the choice of bandwidth. The cross-validation method revealed the optimal bandwidth to be 429 m. The locations of the boreholes identified by the CDC are marked as crosses.

**Figure 6:**
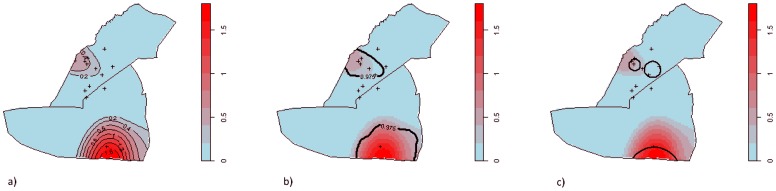
Geographical distribution of the log relative risk (bandwidth  = 429 meters) of typhoid fever in Dzivaresekwa and Kuwadzana suburbs in Harare, Zimbabwe, October 10 2011–March 17 2012, with: a) contours of risk gradients; b) location of clusters identified by random labelling; c) location of clusters identified by Kulldorff's spatial scan technique. The graphs are showing areas with increased risk of typhoid fever (log relative risk over 0), all areas with log relative risk equal or under 0 are represented in blue.

Fig. 6a shows the log relative risk of finding case-patients compared to that of finding control points throughout the epidemic, with the optimal bandwidth selected by the cross-validation method, with contours showing disease log relative risks. Random labelling identified two clusters of disease risk: one each in Dzivaresekwa and Kuwadzana (Fig. 6b). The Kuwadzana cluster had a greater peak log relative risk, an over 1.8-fold increase (relative risk  = 6.1), than the peak of 0.6 (relative risk  = 1.8) in Dzivaresekwa.

Clusters were identified in a similar geographical range by both Kulldorff's spatial scan statistic and random labelling (Fig. 6c).

S1 Video is a video file showing the weekly evolution of the log relative risk of typhoid fever. It confirms graphically that the epidemic was concentrated in the first weeks around certain boreholes in Dzivaresekwa and subsequently shifted to the areas surrounding the boreholes in Kuwadzana.

## Discussion

We found significant spatiotemporal clustering of typhoid fever case-patients during this epidemic. Cluster analysis identified two areas with significantly elevated risk: one each in Dzivaresekwa and Kuwadzana. The spatial scale at which typhoid fever clustered was highly localised, with significant clustering at distances up to 4.5 km and peak levels at approximately 3.5 km, although the focus shifted from the Dzivaresekwa to the Kuwadzana cluster during the epidemic. We identified areas of high risk, and the results provided evidence in support of spatially targeted epidemic response activities, which included water and sanitation interventions (particularly chlorination of boreholes), health education, and hygiene promotion.

Proximity to water sources is a possible reason for the clustering observed; however, the role of other risk factors cannot be excluded, particularly as we were unable to explore these in a case-control study (because of a rapid decrease in the incidence rate of typhoid fever during the study period).

An inverse association between risk of infection and age was observed, which is typical of typhoid-endemic areas [18]. A more even distribution of risk across age groups, suggesting epidemic transmission of typhoid fever, has been reported elsewhere [6], [8]. The typhoid incidence was higher among females, suggesting a greater risk of exposure through household activities, including preparation of contaminated food and caring for children (a high risk group for *S.* Typhi infection).

The principal limitation of this study is that we were unable to establish associations with risk factors other than age, gender and location. In particular, it would have been useful to demonstrate a direct epidemiological link between the epidemic and the boreholes suspected of being the source of contamination. Water samples taken during the epidemic from boreholes close to the clusters were analysed at a laboratory [22], and several contained evidence of faecal contamination. The bacteria could not be isolated or cultured, but *S*. Typhi is notoriously difficult to culture [6], [7], and high levels of faecal contamination in areas of elevated risk for typhoid fever infection is frequently taken as a proxy for *S*. Typhi contamination.

Our method for generating control points does not inherently account for a heterogeneous population distribution within inhabited areas, leading to the possibility that the clustering detected was due at least in part to this heterogeneity. However, within these suburbs, the vast majority of residential structures were observed to be one-story structures housing a single family. Therefore, we believe that such heterogeneity in the population distribution within inhabited areas was not substantial, and that the majority of the clustering observed was therefore due to heterogeneity within spatial patterns of disease risk.

The borehole locations included in the spatial analysis were from a convenience sample of water sources examined by CDC during the epidemic. A more robust method would have been to include the locations of all boreholes in the two suburbs and to compare the relative risks of infection at each location.

Only a small proportion of case-patients (1.2%) had a laboratory-confirmed diagnosis, potentially introducing selection bias, particularly as typhoid fever has several non-specific symptoms in the early phases [2], [38]. However, the identification of strong clustering, despite any ‘noise’ created by the inclusion of false-positive case-patients underlines the power of this method for detecting spatial clusters. Nevertheless, a robust, appropriate definition of suspected cases should be incorporated into the epidemic response efforts [13], [38], [39].

The spatial analysis did not take into account clustering at household level: all traceable case-patients were included in the analysis, including multiple cases within households. While this may affect our results in terms of identifying sources of primary transmission, an advantage is that the results are not limited to clustering due to residential location alone, additionally including clustering due to within-household transmission, which better reflects the actual risk of infection [40]. In addition, secondary case-patients accounted for just 5.3% of the total and therefore had a minimal influence.

As the line list was maintained at health facilities, it is possible that the clustering detected was merely a reflection of underlying spatial patterns of access to these facilities. The observed differences in age-specific attack rates may also have been due to differences in age-specific health-seeking behaviour, e.g. children may be more likely to be taken to a health facility. The inclusion of patients for non-related diseases as controls could have minimised such potential biases [26], [41]; however, because the clustering was so localised and because access to facilities was free at the point of use during this epidemic, geographical and financial barriers probably did not play an important role.

The greatest strength of this study is the intense clustering detected, even after adjustment for the underlying population distribution. The cluster locations were confirmed by two independent methods. Further, the choice of bandwidth, which can affect the results [27], [31], [42], did not modify their location or alter the interpretation of the findings. Our sample size was large and included a high proportion of the line-listed case-patients, giving the analysis power. Spatial clustering of the untraceable case-patients would, however, have introduced some selection bias.

The degree to which typhoid fever clusters spatially has been addressed in only a few studies. In Turkey, the incidence of typhoid fever was elevated in areas of low socio-economic status, a risk factor being contamination of water supplies with sewage [18]. In Viet Nam [6], Nepal [7] and India [9], [12], the disease was spatially associated with proximity to public water sources, Dewan et al. [9] also reporting a spatial association with low elevation. Extensive clustering of typhoid fever independent of population size and density has been described in both Nepal [7] and Malaysia [17], with significant clustering up to distances of 6 km in the latter study. In northern Viet Nam, spatial clusters of cases were considered to be maintained by repeated contamination of water sources by typhoid carriers [6].

Our study adds experience from an urban sub-Saharan African setting, the area estimated to have the greatest incidence of typhoid fever globally [4], [43] and where the disease is re-emerging under the challenges of increasing urbanisation, chronic underinvestment in water and sanitation infrastructure, and emerging drug-resistance [9], [10], [18], [44]. The most robust methods of identifying local clusters are based on point data (as opposed, or in addition, to aggregated data) and take into account the heterogeneity in the underlying population distribution: in this article, we have reported a combined use of these methods to investigate clustering of typhoid fever.

We identified clustering of typhoid fever case-patients at distances up to 6 km, with a peak at around 3.5 km, which is almost identical to the scale of clustering reported in a study in Malaysia [17], suggesting that the scale of clustering of this disease is consistent in different settings. More localised clustering of typhoid fever cases has also been reported, however, Hinman et al. [19] having reported clusters at between 150 m and 1 km.

Our study suggested a possible role of boreholes located within the clusters in the transmission of typhoid fever, suggesting that long-cycle (outside the household), rather than short-cycle (within household), transmission predominated in this epidemic, a finding reported in other studies [7], [12]. These transmission dynamics were substantiated for both typhoid fever and cholera in other studies by an association with water spout proximity and low elevation [7], [9], [45]. In contrast, Shah et al. [8] reported that clusters of epidemic typhoid fever in Kelantan, Malaysia, were unrelated to areas of flood risk or to treatment of water and that short-cycle transmission via contacts or contaminated food played the dominant role.

Improvements in water and sanitation infrastructure and in hygiene conditions are essential for the control and elimination of typhoid fever [2], [3], [5], [7]. Our results, combined with those for recent epidemics of enteric infection in Harare, can be used to advocate for such improvements and for typhoid preparedness efforts in the worst affected areas [16]. As water and sanitation improvements will require considerable investments in time and resources, however, preparedness plans must be developed, as epidemics will continue to occur in the short-to-medium term. Tran et al. [6] suggested that, in resource-poor areas, improving water quality through targeted health education campaigns is the most realistic approach to preventing typhoid fever transmission. The results of our study and similar spatial analyses could be incorporated into, and help guide, such plans.

This study was largely conducted *a posteriori*, nearly 6 months after the onset of the epidemic, thereby limiting its usefulness for identifying geographical areas for targeting prevention and control activities. The analysis could have been performed months earlier with data for a fraction of the case-patients, as it was apparent in the first week of the epidemic that the cases clustered around certain boreholes in Dzivaresekwa (see S1 Figure). Similar analyses should be conducted earlier in the course of epidemics, and, as suggested by Odoi et al. [45], systematic cluster investigation techniques should be incorporated into regular surveillance activities. Classical case-control studies and environmental sampling to determine context-specific risk factors and to describe the relative roles of short-cycle and long-cycle transmission should augment the spatial analytical component of risk factor studies.

In conclusion, we identified clusters of case-patients, a critical step in typhoid control, highlighting the importance of spatial analysis for making timely decisions about where to target prevention and control activities and reinforce treatment during epidemics. When conducted in real time during the onset and progression of an epidemic, such techniques facilitate monitoring of progression and enhance understanding of the transmission dynamics, which in turn enhance the design, implementation and evaluation of intervention strategies.

The techniques described here are powerful tools that complement more classical approaches during epidemics, including laboratory confirmation and case-control studies for investigating risk factors of infection. Surveillance data combined with geospatial techniques can be used to identify priority areas in which thorough epidemiological investigations should be conducted to identify determinants and assess the burden of disease.

## Supporting Information

S1 Figure **Geographical distribution of the log relative risk (bandwidth  = 429 meters) of typhoid fever in Dzivaresekwa and Kuwadzana suburbs in Harare, Zimbabwe, October 10–16 2011.**
(EPS)

S1 Supporting Information **R code for sequential generation of random control points within a polygon shapefile.**
(R) Click here for additional data file.

S1 Video **Animation of the geographical distribution of the weekly log relative risk (bandwidth  = 429 meters) of typhoid fever in Dzivaresekwa and Kuwadzana suburbs in Harare, Zimbabwe, October 10 2011–March 17 2012.**
(M4V)
